# “What Makes Us Strong?”: Dyadic Coping in Italian Prospective Adoptive Couples

**DOI:** 10.3389/fpsyg.2019.00399

**Published:** 2019-03-06

**Authors:** Elena Canzi, Silvia Donato, Laura Ferrari, Miriam Parise, Ariela Francesca Pagani, Giulia Lopez, Rosa Rosnati, Sonia Ranieri

**Affiliations:** ^1^Family Studies and Research University Centre, Università Cattolica del Sacro Cuore, Milan, Italy; ^2^Department of Psychology, Family Studies and Research University Centre, Università Cattolica del Sacro Cuore, Milan, Italy; ^3^Department of Psychology, Family Studies and Research University Centre, Università Cattolica del Sacro Cuore, Piacenza, Italy

**Keywords:** prospective adoptive couples, dyadic coping, relationship satisfaction, couple generativity, actor partner interdependence model

## Abstract

Becoming an adoptive parent is a particularly stressful transition, given the additional challenges couples have to face. Dyadic coping, an under-investigated dimension in the adoption literature, may play a relevant role for prospective adoptive couples’ ability to better cope with the adoptive process. The general aim of the present study was to investigate the association between dyadic coping and relationship functioning, in terms of relationship satisfaction and couple generativity, among prospective adoptive couples. Participants were 103 prospective adoptive couples pursuing international adoption in Italy. Couples were asked to fill in a self-report questionnaire. Results of the Actor-Partner Interdependence Model showed that prospective adoptive partners reported high levels of positive and common dyadic coping and low levels of negative dyadic coping – suggesting partners’ ability to successfully cope together with a common stressor – a high level of relationship satisfaction, and an average level of couple generativity. Moreover, analyses showed significant actor effects of one’s own perception of the partner’s dyadic coping (positive, negative, and common) on one’s own relationship satisfaction and on couple generativity for both wives and husbands. With regard to partner effects, we found that both partners’ perceptions of the other’s dyadic coping responses (positive, negative, and common) were associated with the other’s relationship satisfaction, with the only exception of wives’ perceptions of common dyadic coping, which were not associated with their husbands’ relationship satisfaction. As for couple generativity, the only significant partner effect referred to negative dyadic coping responses for both wives and husbands.

## Introduction

### Prospective Adoptive Couples: Stressors and Resources

Becoming parents is a crucial family transition associated with significant relational, psychological, and social changes. Prospective adoptive couples have to face specific challenges and tasks that make them particularly vulnerable to stress (Canzi et al., 2017b). Most prospective adoptive couples, for example, faced infertility (Cohen et al., 1993; Daniluk and Hurtig-Mitchell, 2003), struggling with the related elaboration process. In addition, they are going through the assessment procedures to obtain adoption suitability, interfacing with bureaucratic systems, which are very demanding and stressful (Palacios and Sánchez-Sandoval, 2006). On the psychological side, moreover, during the pre-adoption phase, they could experience the anticipatory stress related to concerns about the child and the first encounter with him/her; they also have to prepare to legitimize each other as parents of a child “born by others,” the so-called entitlement process (Cohen et al., 1996). Prospective adoptive partners often become parents late in life (in Italy in 2015, the average age for the husband was 45.8 years and for the wife was 44.1 years), after a long waiting period (3 years and 7 months on average in Italy in 2015) (Commission for Intercountry Adoption, 2014/2015), and they expect to cope with children who are likely to be emotionally and behaviorally compromised at arrival, due to their past experiences (i.e., abandonment, neglect, institutionalization; Canzi et al., 2018). All these stressors are likely to impact on couples’ psychological well-being (Goldberg et al., 2010) as well as on their future adjustment to parenthood (Salcuni et al., 2015). Indeed, the pre-adoption phase is so demanding that, between 2006 and 2015, only the 68.6% of Italian couples obtaining the decree of suitability have given a mandate to the adoption agency; among them, only the 66.4% has completed the entire adoption procedure applying for an authorization allowing foreign children to enter Italy (Commission for Intercountry Adoption, 2014/2015). Therefore, more than one-third of the decrees of approval that are issued became ineffective with a great loss of money, resources, and opportunities for these couples as well as for children awaiting adoption. These data suggest that to successfully cope with this demanding pre-adoption phase, couples are required to pool all their individual and relational resources, especially marital ones.

Despite the centrality of this phase of preparation and adaptation for the adoption process, relatively few studies have been conducted on prospective adoptive couples. Most of these studies especially focused on parents’ socio-demographic characteristics, motivations, expectations, ethnic prejudice, and personal well-being. Generally, prospective adoptive couples resulted to have personal resources, in terms of psychological well-being, emotional stability, low levels of ethnic prejudice, and high positive intergroup contacts (Deater-Deckard and Petrill, 2004; Bausch, 2006; Salcuni et al., 2006; Welsh et al., 2008; Zhang and Lee, 2010; Park and Hill, 2014; Canzi et al., 2017a). Much less investigated were their relational and marital resources (e.g., Levy-Shiff et al., 1990). Although research conducted on adoptive parents during later stages of the adoption transition evidenced a global positive quality of adoptive couples’ relationship (Lansford et al., 2001; Ceballo et al., 2004; Rosnati et al., 2013; Canzi et al., 2017b), we do not know much about prospective adoptive couples’ relationship more generally and about their coping ability more specifically. Nonetheless, a study examining adoptive couples relationship quality across the transition to adoption shows that pre-adoptive coping resources represent a protective factor against a pre to post-adoption decrease in satisfaction (Goldberg et al., 2010). Given the peculiar stressors faced by prospective adoptive couples, in fact, partners’ ability to cope jointly against stressful events (i.e., dyadic coping) may be considered as a functional skill that can help to overcome these challenging situations as well as strengthen their relationship (Bodenmann, 1997, 2005).

### Dyadic Coping: A Resource for Couples’ Functioning

Dyadic coping refers to a dyadic process in which partners cope together with stressful circumstances. Among the different conceptualizations of dyadic coping (cfr. Iafrate and Donato, 2012; Acquati and Saita, 2017), we focused here on the Systemic Transactional Model (STM) developed by Bodenmann (1995; 1997; 2000). Within the STM, stress can be conceptualized not only as an individual phenomenon, but also as a dyadic event: Dyadic stress refers to those circumstances that affect (either directly or indirectly through the other partner’s stress) both members of the couple and elicit joint appraisal of the situation as well as common coping responses to it (Bodenmann, 1995; Lyons et al., 1998). Specifically, dyadic coping is a process in which one partner’s communication of stress is perceived, decoded and evaluated by the other, who then responds with his/her coping reactions. Such responses can be either (emotion-oriented or problem-oriented) supportive behaviors one partner enacts toward the other (e.g., one partner showing understanding or offering solutions) or common responses both partners engage in to cope with stress together (e.g., joint problem solving, relaxing together, etc.). The aim of dyadic coping is twofold: It is intended to restore or maintain both partners’ individual well-being, by reducing the partners’ levels of stress, and to promote couple functioning, by strengthening partners’ sense of we-ness and reciprocal trust (Cutrona, 1996; Bodenmann, 2005). Dyadic coping styles, however, can also be ineffective or unskillful (i.e., the so-called negative dyadic coping). When this is the case, the coping process risks to be unsuccessful and the relationship undermined (e.g., Donato and Parise, 2012; Falconier et al., 2015). Differently from the other conceptualizations of dyadic coping, that were elaborated mostly within the context of chronic illness, Bodenmann’s theory was first developed to define coping with daily hassles (minor stressors; Donato et al., 2015). Only later it was extended to critical life events (major stressors), such as acute and chronic illness (e.g., Revenson and DeLongis, 2010; Bertoni et al., 2015; Traa et al., 2015), couples’ coping with normative transitions (e.g., transition to marriage, Donato et al., 2012; couples’ aging, Landis et al., 2013) as well as non-normative ones (e.g., couples’ facing the death of a child, Bergstraesser et al., 2015; couples dealing with a child with autism, García-López et al., 2016; step-family formation, Lee-Baggley et al., 2005).

Only one study, to our knowledge, analyzed dyadic coping in the context of adoption (Hock and Mooradian, 2012). This study examined the contributions of individual and relational characteristics (dyadic coping, dyadic adjustment, and conflict resolution styles) to the quality of adoptive mothers’ co-parenting and found that higher levels of positive dyadic coping were associated with better co-parenting. Moreover, dyadic coping was a stronger predictor of co-parenting quality than conflict resolution and marital quality. This study, however, could be usefully extended in three respects. First, the study focused on the post-adoption phase rather than on the pre-adoption one. Second, Hock and Mooradian’s (2012) study tested the effects of dyadic coping on adoptive mothers, rather than focusing on the couple as a whole. In dyadic coping, however, both partners are engaged and influential on one another. In addition, within the transition to adoptive parenthood, both partners are strongly involved and engaged from the very beginning of the process. In particular, adoptive fathers seem to represent a crucial resource for the adjustment to adoption and for children lifespan development (Ferrari et al., 2015; Ranieri et al., 2017), so that some authors have spoken of an “adoptive-enhanced fatherhood” (Levy-Shiff et al., 1997). A dyadic approach to studying dyadic coping within these couples is therefore particularly warranted. Third, Hock and Mooradian’s (2012) study focused on the role of dyadic coping for adoptive mothers’ parenting skills. The role that dyadic coping plays for prospective adoptive couples’ marital quality is as important as its effects on their parenting skills for at least two reasons. On the one hand, given that marital functioning prior to adoption is related to adoptive parents’ risk of relationship dissolution (e.g., Goldberg and Garcia, 2015), it is worth investigating those factors that can foster these couples’ relationship. On the other, given the links between marital quality and parenting skills, well-documented in the general population (Erel and Burman, 1995; Margolin et al., 2001; Stone et al., 2002; Bradford et al., 2003; Stright and Bales, 2003), examining predictors of prospective adoptive couples’ marital functioning would also suggest a potential way to indirectly promote their parental competences.

### The Present Study

The goal of this study was to investigate the association between dyadic coping and relationship functioning among Italian prospective adoptive couples. Specifically, we focused on two different aspects of relationship functioning: Relationship satisfaction and couple generativity. While research generally identifies as one of the main outcomes of couple relationship with relational satisfaction, couple generativity is relatively under-investigated. A well-known psychological theory by Erikson (1963) postulates that for the adult individual the most important developmental outcome is not the achievement of a mere well-being, rather it refers to the unfolding of his/her capacity of being “generative.” Generativity is the ability to move away from a narcissistic self-concern to take care of those who are to follow (Erikson, 1963; McAdams et al., 1993). Generativity does not refer exclusively to procreation (i.e., the biological level), but may be expressed also at the social level, by taking care of future generations through teaching, mentoring, political engagement as well as engagement with youth protection and health. Inspired by this theory, some scholars (Cigoli and Scabini, 2006; Parise et al., 2017) have started to argue that the good functioning of a relationship cannot be measured only in terms of relationship satisfaction, but it may involve also the ability to go beyond one’s boundaries as a couple and to take care of social bonds. Couple generativity seems a relevant component of prospective adoptive couples’ functioning, as adoption in itself can be considered a form of social generativity (Cigoli and Scabini, 2006; Scabini and Rossi, 2014). Research on community couples has found that couple generativity is related to partners’ trust, intimacy, commitment, and romantic affect (Bertoni et al., 2012), but no studies investigated generativity (nor the role of dyadic coping for it) in prospective adoptive couples.

## Materials and Methods

### Participants and Procedure

Participants were 103 heterosexual prospective adoptive couples living in the North of Italy. Couples were contacted in the process of completing international adoptions, before the actual arrival of the child. All partners (*N* = 206 individuals) were married. The exclusion criterion was having already one or more children at the time of the study. Wives’ average age was 40.2 (*SD* = 4.1), ranging from 29 to 46 years, and husbands’ average age was 41.8 (*SD* = 4.4), ranging from 29 to 57 years. Sixty-two point one percent of couples have resorted to assisted reproductive technology, on average 3.44 (*SD* = 2) times (range 1–8). The average duration of marriage was 8.4 years (*SD* = 4.2) and ranged from 1 to 19 years. All couples had attained a medium-high level of education: 54% of wives and 57% of husbands had up to 13 years of education, while the remainder had studied for 16 years or more. All participants were recruited through advertisements placed in different venues and contexts (e.g., schools, family associations, and adoption agencies) and through snowball sampling. Participants were given two self-report questionnaires, one for the wife and one for the husband, and were asked to complete their respective questionnaire independently from their partner. Anonymity and data confidentiality were guaranteed. All participants took part in the study voluntarily and gave informed and written consent. The study protocol was not reviewed by the ethics committee, since it was not required at the time of data collection, according to the local and national guidelines. However, it followed the standard ethical guidelines of the Italian Association of Psychology (AIP) and the standard ethical guidelines of the American Psychological Association (APA).

### Measures

The instrument used was a self-report questionnaire composed of the following scales.

#### Dyadic Coping

To measure dyadic coping we used the Dyadic Coping Questionnaire (Fragebogen zur Erfassung des Dyadischen Copings als stabile Tendenz; FDCT-N, Bodenmann, 1997; Donato et al., 2009). This scale is composed of 41 items on a 5-point scale (from 1 = *never* to 5 = *very often*) and measures the processes involved in dyadic coping, including stress communication, dyadic coping responses, and satisfaction with dyadic coping (Bodenmann, 1995, 1997, 2005). In this study, we considered the subset of items referring to the perceptions of the other’s dyadic coping responses. First, we assessed perceptions of the other’s positive dyadic coping (seven items), that is the extent to which the responses of the partner to one’s own stress are supportive. Sample item is: “When I am stressed, my partner shows me his/her interest and understanding.” Second, we assessed perceptions of the other’s negative dyadic coping (five items), that is, the extent to which the partner’s responses to one’s own stress are perceived as negative. Sample item is: “My partner makes fun of my stress and mocks me.” Third, we assessed perceptions of common dyadic coping (seven items), that is how both partners respond to communicated stress. Sample item is: “We try to cope with the problem together and search for practical solutions.” We created a global index of positive dyadic coping responses (α = 0.74 wives and α = 0.72 for husbands), a global index of negative dyadic coping responses (α = 0.53 wives and α = 0.58 for husbands), and a global index of common dyadic coping responses (α = 0.76 wives and α = 0.72 for husbands) by averaging the corresponding items. A higher score indicated a higher level of the corresponding dyadic coping response.

#### Relationship Satisfaction

To measure relationship satisfaction we used the Quality of Marriage Index (Norton, 1983). The scale is a six-item inventory: The first five items (e.g., “The relationship with my partner makes me happy”) are on a 7-point scale (1 = *completely disagree*, 7 = *completely agree*), whereas the last item, measuring a global perception of relationship satisfaction, is on a 10-point scale (1 = *very unhappy*, 10 = *very happy*). We used the first five items and averaged them to a global index of satisfaction (α = 0.91 for wives and α = 0.90 for husbands). A higher score indicated a higher level of relationship satisfaction.

#### Couple Generativity

Couple generativity was assessed through the Couple Generativity Scale (Parise et al., 2017), which is composed of four items on a 9-point scale (from 1 = *completely disagree* to 9 = *completely agree*). Items are: “We are committed as a couple to our community”; “We are a reference point for our friends”; “We think that our experience as a couple can be made available to other people”; “Our friends often asks for advice to us as a couple.” The items were averaged to form a global index of couple generativity and showed good internal consistency (α = 0.77 wives and α = 0.76 for husbands). A higher score indicated a higher level of couple generativity.

### Data Analyses

To deal with data interdependence, we used the actor-partner interdependence model (APIM, Kenny, 1996; Kenny and Cook, 1999) for testing the association of dyadic coping responses (positive, negative, and common) with relationship satisfaction and couple generativity. The APIM is a dyadic data analytic approach that treats the couple as the unit of analysis. That is, the APIM estimates effects for both members of the couple simultaneously, while controlling for their interdependence (Kenny et al., 2006), and tests the interpersonal effects of one couple member’s report on one’s own (i.e., actor effect) and on the other member’s (i.e., partner effect) outcome. We tested both actor effects and partner effects using the software AMOS 22. Finally, to examine gender differences, women’s and men’s paths of interest in the model were constrained to be equal and the χ^2^ difference test was performed. In case the constrained model showed no significantly different fit from the unconstrained one, the constrained, more parsimonious model was retained. In the figures, whenever no gender differences emerged, we presented pooled coefficients across genders as final estimates of the empirical models.

## Results

### Descriptives

The sample was composed of partners that generally reported to perceive the other as highly supportive (i.e., providing a high level of positive dyadic coping and a low level of negative dyadic coping), to successfully cope together with a common stressor, showing similar or slightly better dyadic coping abilities than reported in other Italian samples (Donato et al., 2015, 2018; Parise et al., 2018). Moreover, they reported high levels of relationship satisfaction, and average levels of couple generativity (see Table 1). As for gender differences, wives reported higher levels of common dyadic coping [*t*(102) = 2.86, *p* = 0.005] and couple generativity [*t*(102) = 2.94, *p* = 0.004] than husbands. No other significant gender differences emerged. Correlations between dyadic coping indexes as well as between dyadic coping and outcomes were as expected (see Table 1). In particular, in both wives and husbands, positive and common dyadic coping were positively correlated with each other and negatively with negative dyadic coping. Again in both wives and husbands, positive and common dyadic coping were positively associated with both relationship satisfaction and couple generativity, while negative dyadic coping was negatively correlated with the above outcomes. Relationship satisfaction and couple generativity were positively correlated with each other, but correlations were low to moderate in size, thereby suggesting that the two constructs were not overlapping.

**Table 1:** Correlations, means, and SD of the variables of the study.

Variable	1	2	3	4	5	*M*	*SD*
1. Positive dyadic coping	**0.31^∗∗∗^**	-0.43^∗∗∗^	0.51^∗∗∗^	0.48^∗∗∗^	0.39^∗∗∗^	3.93	0.59
2. Negative dyadic coping	-0.46^∗∗∗^	**0.35^∗∗∗^**	-0.27^∗∗^	-0.42^∗∗∗^	-0.27^∗∗^	1.27	0.32
3. Common dyadic coping	0.44^∗∗∗^	-0.25^∗^	**0.50^∗∗∗^**	0.54^∗∗∗^	0.40^∗∗∗^	3.91	0.54
4. Relationship satisfaction	0.35^∗∗∗^	-0.37^∗∗∗^	0.41^∗∗∗^	**0.56^∗∗∗^**	0.41^∗∗∗^	6.51	0.70
5. Couple generativity	0.21^∗^	-0.21^∗^	0.26^∗∗^	0.24^∗^	**0.70^∗∗∗^**	5.01	2.00
*M*	3.91	1.22	3.74	6.53	4.57		
*SD*	0.51	0.33	0.63	0.62	1.87		

*N = 103 couples. Correlations for husbands appear below the diagonal; Correlations for wives appear above the diagonal. Boldface values along the diagonal are correlations between husbands-wives dyad members. Means and standard deviations for wives appear in the vertical columns. Means and standard deviations for husbands appear in the horizontal columns; ^∗^*p* < 0.05, ^∗∗^*p* < 0.01, ^∗∗∗^*p* ≤ 0.001*.

### Associations Between Perceptions of Dyadic Coping Responses and Relationship Satisfaction

**Figure 1:**
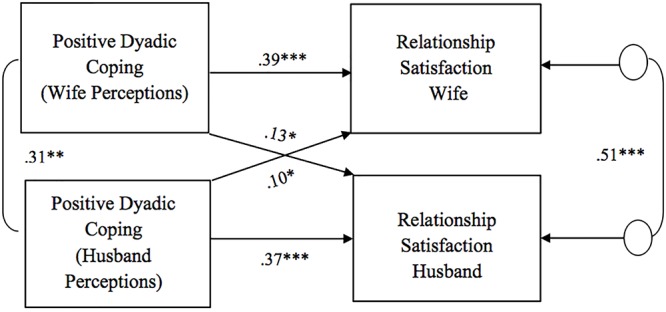
Associations between perceptions of positive dyadic coping responses and relationship satisfaction. Path coefficients are standardized estimates; ^∗^*p* = 0.05; ^∗∗^*p* < 0.01; ^∗∗∗^*p* ≤ 0.001.

**Figure 2:**
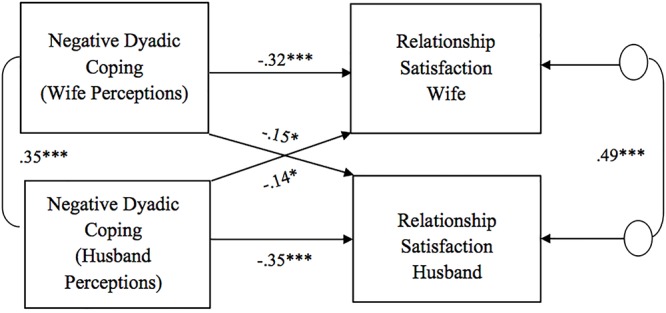
Associations between perceptions of negative dyadic coping responses and relationship satisfaction. Path coefficients are standardized estimates; ^∗^*p* < 0.05; ^∗∗∗^*p* ≤ 0.001.

**Figure 3:**
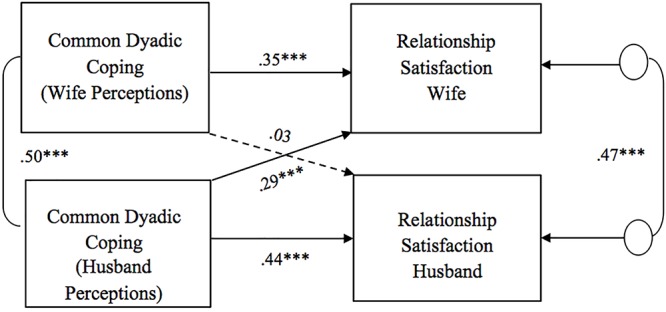
Associations between perceptions of common dyadic coping responses and relationship satisfaction. Path coefficients are standardized estimates; ^∗∗∗^*p* ≤ 0.001.

As for the association between perceptions of dyadic coping responses (positive, negative, and common) and relationship satisfaction, all models showed significant actor effects of one’s own perception of the partner’s dyadic coping on one’s own relationship satisfaction for both wives and husbands (Figure 1–3). That is, one’s perceptions of the other as supportive (i.e., positive DC) as well as the couple as a good team in coping with stress (i.e., common DC) were positively associated with one’s own relationship satisfaction. One’s perceptions of the other as unsupportive (i.e., negative DC) were negatively related to one’s own relationship satisfaction. No gender differences were found in actor effects. With regard to partner effects, we found that both partners’ perceptions of dyadic coping responses also predicted the other partner’s relationship satisfaction, with the only exception of wives’ perceptions of common dyadic coping which were not associated with their husbands’ relationship satisfaction. Specifically, partners’ perceptions of the other as supportive were positively related to the other’s relationship satisfaction, while -on the contrary- partners’ perceptions of the other as unsupportive were negatively related to the other’s relationship satisfaction (Figure 1, 2). No gender differences were found in the above partner effects, while partner effects related to the common dyadic coping model were significantly different between husbands and wives (Figure 3). Specifically, while husbands’ perceptions of common dyadic coping were positively associated with their wives’ relationship satisfaction, wives’ perceptions were not.

### Associations Between Perceptions of Dyadic Coping Responses and Couple Generativity

**Figure 4:**
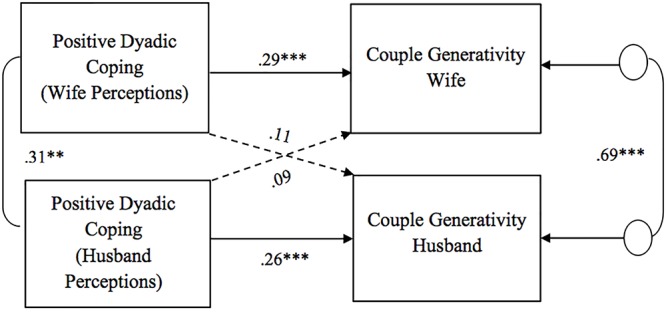
Associations between perceptions of positive dyadic coping responses and couple generativity. Path coefficients are standardized estimates; ^∗∗^*p* < 0.01; ^∗∗∗^*p* ≤ 0.001.

**Figure 5:**
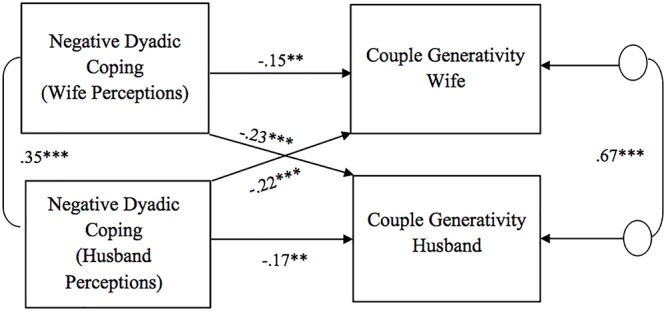
Associations between perceptions of negative dyadic coping responses and couple generativity. Path coefficients are standardized estimates; ^∗∗^*p* < 0.01; ^∗∗∗^*p* ≤ 0.001.

**Figure 6:**
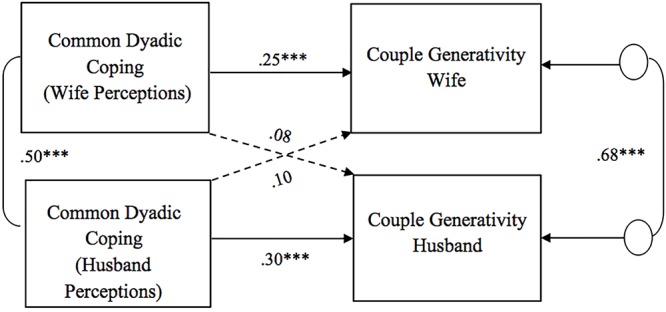
Associations between perceptions of common dyadic coping responses and couple generativity. Path coefficients are standardized estimates; ^∗∗∗^*p* ≤ 0.001.

As for the association between perceptions of dyadic coping responses (positive, negative, and common) and couple generativity, all the APIM models showed significant actor effects for both husbands and wives (Figure 4–6). Specifically, partners’ perceptions of the other as supportive and of the couple’s positive common efforts to cope with stress were positively related to their own couple generativity, while partners’ perceptions of the other as unsupportive were negatively related to their own couple generativity. No gender differences were found in actor effects. With regard to partner effects, we found that partners’ perceptions of the other’s negative dyadic coping responses predicted the other’s couple generativity (Figure 5). Specifically, partners’ perceptions of negative dyadic coping from their partner negatively predicted their partner’s couple generativity. No gender differences were found in the above association and no other partner effects were detected.

## Discussion

The present study aimed at investigating the perceptions of dyadic coping responses (positive, negative, and common) among Italian prospective adoptive couples, as well as exploring the associations with relationship functioning in terms of relationship satisfaction and couple generativity. Results showed that prospective adoptive couples reported high levels of positive dyadic coping and low levels of negative dyadic coping, suggesting partners’ ability to successfully cope together with a common stressor, a high level of relationship satisfaction, and an average level of couple generativity. In line with the literature (Lansford et al., 2001; Ceballo et al., 2004; Rosnati et al., 2013; Canzi et al., 2017b), adoptive couples resulted to be well-equipped and to have relational resources, especially in terms of couple relationship functioning. It could be that couples choosing adoption are those who can count on a wide range of resources. Such resources may derive from partners’ personal skills or from their good relational adjustment as well as from the experiences related to the adoption transition. Most of these couples, in fact, struggled with many critical events. Several, for example, failed assisted reproductive treatments. We can speculate that, even for couples who were not facing these experiences prior to adoption, going through the difficulties related to the decision to adopt strengthened their bond, encouraged their investment in the couple relationship, and increased their resilience. We can therefore hypothesize that the pre-adoptive period and its challenges can function somehow as a “training” process, that could enhance and promote partners’ competences and resources to face the challenges related to the adjustment to adoption. This possibility is in line with models showing that challenging and stressful life experiences can benefit some couples by improving their resources and contribute to positive growth (Meichenbaum, 1985; Riley, 2013). Future research should test this possibility by evaluating the links between the level of stress experienced in the pre-adoption process, dyadic coping competences, and relationship quality in this type of couples.

Moreover, analyses evidenced that one’s perceptions of the other as supportive (i.e., positive DC) as well as the couple as a good team in coping with stress (i.e., common DC) were positively associated with one’s own relationship satisfaction and couple generativity. On the contrary, one’s perceptions of the other as unsupportive (i.e., negative DC) were negatively related to one’s own relationship satisfaction and couple generativity. Relationship satisfaction resulted to be sensitive to partner effects as well and therefore be associated to the other’s perceptions of one’s own dyadic coping responses, with the only exception of wives’ perceptions of common dyadic coping that were not related to husbands’ relationship satisfaction. Couple generativity was also predicted by the other’s perceptions of one’s own negative dyadic coping, while no partner effects were found for positive and common dyadic coping responses.

These findings reveal in both wives and husbands the presence of significant actor effects on relationship satisfaction and couple generativity. With regard to relationship satisfaction, these results are in line with the literature on dyadic coping in other populations (e.g., Donato et al., 2015; Hilpert et al., 2016) and suggest that, when positive, dyadic coping is a relevant resource for this kind of couples. Feeling supported by the other in times of stress, and feeling that both are engaged in dealing with the problem, promotes prospective adoptive partners’ relationship satisfaction, while perceiving the other as hostile or ambivalent in stress management may undermine their relationship satisfaction. These findings extend the literature on the consequences of dyadic coping in two respects. First, they confirm the role of dyadic coping also for prospective adoptive couples. Second, findings related to couple generativity show that resources that are internal to the couple, such as dyadic coping, allow partners to go beyond themselves and their couple relationship. The way partners are able (or not able) to take care of each other in times of stress seems to spill over to their ability to care for others beyond the couple. This spillover effect seems especially important when partners become parents (Zemp et al., 2016, 2017) and may be crucial for adoptive partners.

The present findings also show significant partner effects. In particular, the perceptions the other holds about one’s own dyadic coping responses are associated with one’s relationship satisfaction, thereby confirming the interdependent and dyadic nature of the dyadic coping process. Dyadic coping, in fact, is a process in which both partners are involved and in which both partners’ individual and relational well-being is at stake (Bodenmann, 1995, 2005). For adoptive couples, moreover, both partners’ involvement is especially required in the transition: assessment procedures put both partners in the spotlight and when the child arrives both partners are involved to the same extent from the very beginning at his/her arrival and along his/her development (Levy-Shiff et al., 1997; Ferrari et al., 2015; Ranieri et al., 2017). With regard to common dyadic coping, only wives’ relationship satisfaction is subject to a partner effect. While wives’ satisfaction is sustained by both their own and their husbands’ perceptions of common dyadic coping, that is their perceptions of common, couple-level efforts to deal with stress, husbands’ satisfaction is promoted by their own perceptions of common dyadic coping only. This finding could be explained by women’s relational orientation (Cross and Madson, 1997): especially when referring to partners’ perceptions of the couple as a whole, women seem affected by their own and their partners’ feelings. Men, on the contrary, being more independence-oriented (Cross and Madson, 1997), may rely more on their own perceptions. On couple generativity the only significant partner effect refers to negative dyadic coping responses. The other’s perceptions about one’s own dyadic coping responses as hostile, distant, or ambivalent, that is the other’s perceptions about one not investing in the relationship and being destructive in times of stress, undermine one’s ability to be generative. It seems that, to be generative as a couple, it is important that partners refrain from destructive responses and actually invest their resources in favor of the other and of the couple relationship. Noticeably, if one partner is perceived as destructive for the relationship, this also impedes him/her to use the relationship as a resource for others. The literature on dyadic coping has testified the detrimental consequences of perceived negative dyadic coping on community couples’ relationship satisfaction (e.g., Donato and Parise, 2012). This study extends previous findings to prospective adoptive couples and to couple generativity, especially showing that not only actor effects of perceived negative dyadic coping, but also partner effects are relevant for both relationship satisfaction and couple generativity.

The present findings bear also practical relevance for preventive and supportive interventions with prospective adoptive parents. In particular, this study highlights the importance of sustaining prospective adoptive parents’ ability to be a good team in coping and facing together with a common stressor, in order to improve their relationship quality as well as their willingness to take care of others and to promote the well-being of young generations and of the society. This could, in turn, contribute to enhance parental competencies and to create a positive and collaborative family climate.

The validity and implications of the present findings should be considered in light of some limitations. First, our sample size is small, so caution is needed when generalizing our findings to the whole population of prospective adoptive parents. Second, we are unable to draw causal inferences, due to our correlational design. Future longitudinal studies could help confirm the direction of effects as well as explore the role of dyadic coping in the post-adoption phase. A final limitation has to do with the exclusive reliance on self-reports. Further research could rely on daily and observational measures in order to deeply capture the complexity of marital functioning.

Despite these limitations, our study, highlights the relevance of extending the investigation of dyadic coping to couples in the pre-adoption phase. Our results, in fact, clearly show that dyadic coping is a crucial resource for prospective adoptive couples’ relationship, which may help them better face the challenges related to adoption.

## Author Contributions

All authors equally contributed to the development of the theoretical framework, to the performance of the statistical analyses, to the analysis of the results, and to the writing of the manuscript.

## Conflict of Interest Statement

The authors declare that the research was conducted in the absence of any commercial or financial relationships that could be construed as a potential conflict of interest.
